# Emulation of Brain Metabolic Activities Based on a Dynamically Controllable Optical Phantom

**DOI:** 10.34133/cbsystems.0047

**Published:** 2023-09-13

**Authors:** Yuxiang Lin, Cheng Chen, Zhouchen Ma, Nabil Sabor, Yanyan Wei, Tianhong Zhang, Mohamad Sawan, Guoxing Wang, Jian Zhao

**Affiliations:** ^1^ Department of Micro-Nano Electronics, Shanghai Jiao Tong University, Shanghai, China.; ^2^ Electrical Engineering Department, Faculty of Engineering, Assiut University, Assiut, Egypt.; ^3^ Shanghai Mental Health Center, Shanghai Jiao Tong University School of Medicine, Shanghai, China.; ^4^ CenBRAIN Neurotech, School of Engineering, Westlake University, Hangzhou, China.

## Abstract

This paper presents a dynamic optical phantom for the simulation of metabolic activities in the brain, and a linear equivalent model is built for control voltage versus substance concentration. A solid–solid dynamic optical phantom is realized by using liquid crystal film as a voltage-controlled light intensity regulator on the surface of basic phantom, which uses epoxy resin as matrix material and nanometer carbon powder and titanium dioxide powder as absorption and scattering dopants, respectively. The dynamic phantom could mimic near-infrared spectrum (NIRS) signals with sampling rate up to 10 Hz, and the maximum simulation errors for oxy-hemoglobin and deoxy-hemoglobin concentrations varying in the range of 1 μmol/l are 7.0% and 17.9%, respectively. Compared with similar solid biomimetic phantoms, the adjustable mimic substance concentration range is extended by an order of magnitude, which meets the simulation requirements of most brain NIRS signals.

## Introduction

Near-infrared spectrum imaging (NIRS) is a promising portable non-invasive brain imaging technique, such as the brain–computer interface (BCI) technique [1–3] and clinical cerebral oxygen monitoring [4–6]. Although NIRS has been widely used by researchers [7], the performances of NIRS devices still need to be improved to provide higher spatial resolution and higher temporal resolution to monitor brain activities more precisely [8–10]. In NIRS device research, especially the performance verification of NIRS measurement systems and imaging detection methods, a series of objects with known optical properties are necessary and important to serve as standard tissue references [2]. Optical phantom is a physical prosthesis that acts as an agent of biological tissue with similar optical properties to biological tissue [11]. Its optical characteristics are stable, controllable, and repeatable. Therefore, it is often used as a standard measure object in new NIRS algorithm verification, NIRS signal testing method verification, and NIRS equipment performance verification and calibration. The main principle of non-invasive near-infrared brain research is to measure changes in absorption coefficient and scattering coefficient caused by brain activities during testing. Thus, optical phantoms to mimic brain tissue should have known absorption and scattering properties, especially for those phantoms that act as benchmarks for NIRS device [12].

A lot of NIRS optical phantoms have been reported in the literature, as different types of optical phantoms are required as suitable measurement objects in different test scenarios. In general, optical phantoms can be divided into 2 categories according to the adjustability of optical parameters: static phantom and dynamic phantom. Static phantoms have fixed optical parameters and are often used as test objects for optical parameter quantification and validation [13,14]; dynamic phantoms have adjustable optical parameters and can be used to simulate dynamic NIRS signals such as tissue optical property changes caused by brain activities [15].

The dynamic performances of NIRS devices and imaging algorithms, such as measurement range, sampling rate, and signal-to-noise ratio, are very important for brain function monitoring since brain activity signals are dynamic. Based on the dynamic phantom, NIRS signals could be simulated by adjusting the absorption and scattering properties. With the controllable and repeatable simulated NIRS signals, the dynamic performance of NIRS systems can be tested better and more advanced NIRS signal detection methods can be explored [11].

There are various physical forms of dynamic phantoms, which can be classified into 4 types according to the physical state (liquid or solid) of the phantom substrate and the regulator: liquid–liquid type [16], liquid–solid type [17,18], solid–liquid type [19,20] and solid–solid type [11,21]. All of these 4 types have advantages and limitations in different applications. (a) The liquid–liquid dynamic phantom adjusts the extinction dopant concentration conveniently by adding liquids with higher dopant concentration into the liquid phantom to increase the optical coefficients, but the disadvantage is that changing the optical parameters in the reverse direction is not that easy. (b) The liquid–solid dynamic phantom changes the optical properties of a certain position of the phantom by placing solid objects with different optical properties in the liquid phantom. It needs to make several solid regulators, and the adjustment process is complicated and slow and cannot be continuously adjusted. (c) The solid–liquid dynamic phantom uses a solid phantom to form a cavity, and the optical properties of the phantom can be adjusted by injecting liquids with different optical properties into the cavity. However, in practice, the liquids must be prepared on site, which poses a substantial obstacle to their use. (d) The solid–solid dynamic phantom adjusts the absorption/scattering coefficient by placing a solid with different optical properties in the solid phantom, which completely gets rid of the liquid material and has the advantages of high stability and long-term storage. Despite the solid–solid phantom’s many benefits, enabling its dynamic properties is difficult. The development of color-changing materials has greatly expanded the range of optical parameter adjustment of solid–solid dynamic phantom, especially the popularity of color-changing materials such as thermochromic materials and electrochromic materials, provides new ideas for solid–solid dynamic phantom, and solves the problem of replacing the optical property regulators in solid–solid dynamic phantom, which is the development trend of dynamic phantom.

Currently, although there are several companies that could provide NIRS phantom, there is still no universal standard for solid-state phantom used in NIRS research, and most NIRS research teams make the phantom that they need for NIRS research by themselves [19,21,22]. In this work, we investigate a novel brain simulation technique based on a dynamic optical phantom, which is based on the solid-state phantom made by using epoxy resin as the substrate and nano carbon powder and titanium dioxide powder as absorption and scattering dopants, respectively, and involves a liquid crystal (LC) film as the voltage-controllable regulator to realize the solid–solid dynamic phantom.

The rest of this paper is organized as follows. The Linear model of NIRS signal for dynamic phantom section describes the basic components and the optical equivalent model of the dynamic phantom. The Design and implementation of optical phantom section explains the processing and implementation of the optical phantom. The Results section presents the test results of the phantom fabricated in this work. The Conclusion section concludes the paper.

## Methods

## Linear Model of NIRS Signal for Dynamic Phantom

**Fig. 1.**
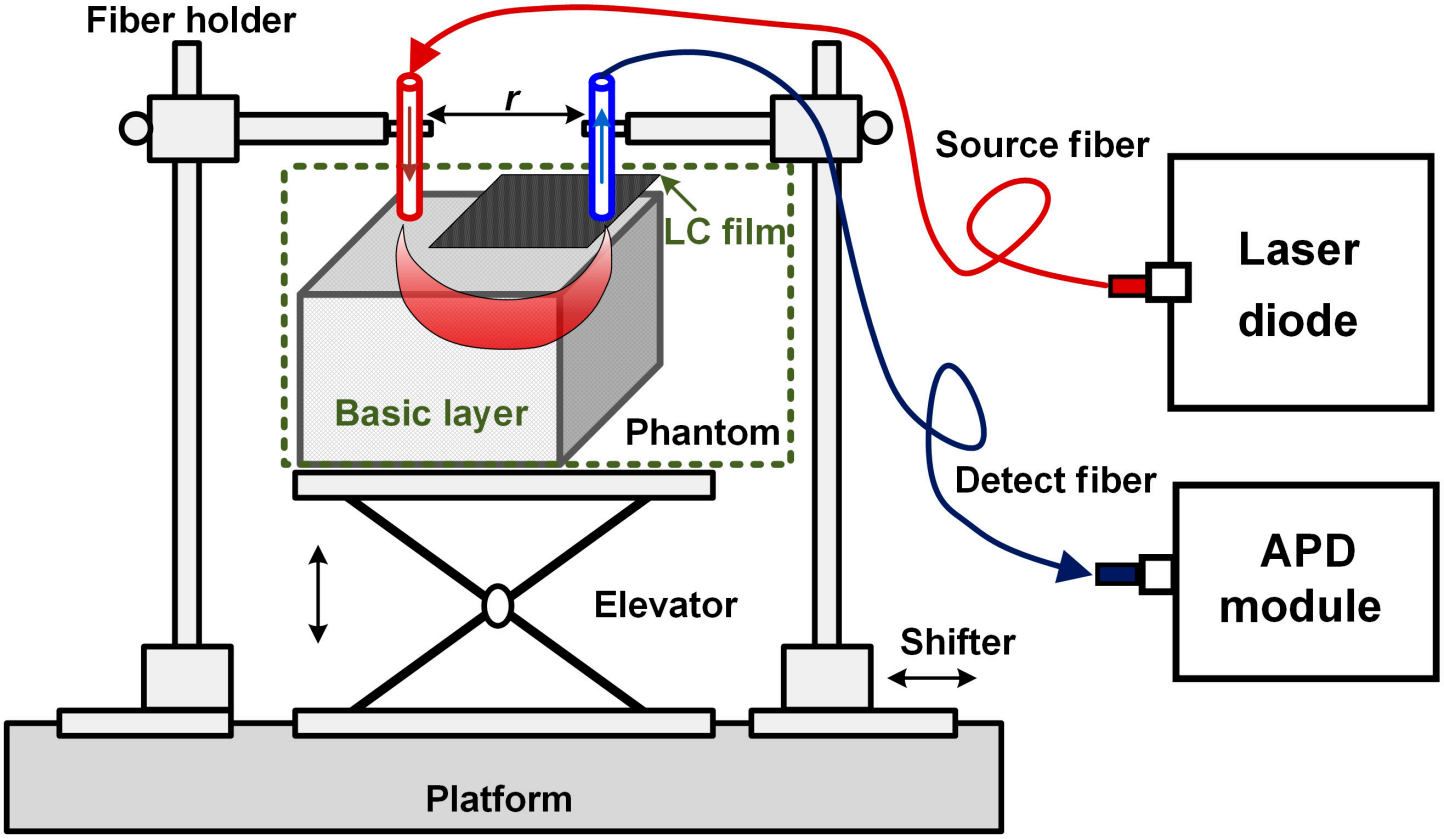
Optical phantom and test platform.

The dynamic optical phantom is composed of a basic layer and a thin LC film layer, which is tightly attached to the top surface of the basic layer, as shown in Fig. 1. In Fig. 1, a typical NIRS device using a solid phantom has been shown. During the test, the phantom is placed on the elevator and fixed to prevent movement. The NIRS device uses a source optode (SO) to emit light and a detection optode (DO) to receive light. The SO and DO use optical fibers to guide light. One side of these 2 optode fibers is held in place by a fiber holder, respectively. The end of the fiber head is pressed against the surface of the phantom and kept vertical, to couple light to the phantom reliably. For SO, the other end of the fiber is connected to the laser and passes the light from the laser onto the phantom surface. The incident light will diffusely propagate and be absorbed in the basic layer. Some of the incident light photons will return back to the top surface after being reflected by scattering particles many times. The light photons that exit from the top surface will pass through the LC film layer and they will be regulated by the voltage on the film before arriving at the DO. The other end of the DO is a photodetector. In this paper, an avalanche photo diode with a large sensing area with integrated high voltage bias and photoelectric-to-voltage converter is used. The output voltage signal can be measured directly by a network analyzer with the frequency domain NIRS method [23].

The light extinction ability of LC film could be adjusted by the electrical field applied on the 2 sides of the film. Obviously, the optical extinction property of the solid–solid phantom is determined by 2 parts: the static part is determined mainly by the basic layer, which consists of resin and pigments; the dynamic part is provided by the LC film with different voltages. In order for the dynamic phantom to simulate the dynamic brain signal comprehensively, it is essential to build a dynamic optical model of the dynamic phantom, i.e., to build the relationship between the optical parameters of the phantom and the voltage. Since the NIRS signals of the brain are primarily a function of oxygenated hemoglobin concentrations and deoxygenated hemoglobin concentrations, it is important to construct a relationship model between LC film control voltage and hemoglobin concentration.

### Optical equivalent model of the dynamic phantom

The light attenuation influence brought by the LC film and its voltage is reflected in the detected light intensity change. In order to construct the equivalent model of LC film voltage versus dynamic phantom optical properties, the way that the Modified Bill Lambert Law (MBLL) [24] deals with the relationship between the change of light intensity and the optical properties of the medium can be used as a reference. Therefore, the attenuation introduced by the film can be equivalent to the optical characteristics of the phantom. This allows us to construct an equivalent model between the voltage and optical characteristics of the dynamic phantom.

The basic absorption coefficient and reduced scattering coefficient of the phantom are respectively *μ*_*a*0_ and $\mu_{s0}^{\prime }$. In addition, the distance between the SO and the DO on the top surface of the phantom is defined by *r*, the light emitted by the light source enters the phantom and propagates diffusely, and some of the photons will be received by the DO. If the light intensity emitted by the source is *I*_0_, and the light intensity received by the DO is *I*_1_, then under semi-infinite boundary conditions, the attenuation can be expressed as Eq. 1 [25],

$$A_{0}=\ln\left(\frac{I_{0}}{I_{1}}\right)=\mu_{\mathrm{eff}\;0}\cdot r$$ (1)

where *μ*_*eff*0_ is the effective attenuation coefficient of the phantom, which can be expressed as Eq. 2 under the semi-infinite boundary condition.

$$\mu_{\mathrm{eff}\;0}=\sqrt{3\mu_{a0}\left({\mu^{\prime }}_{s0}+\mu_{a0}\right)}$$ (2)

The attenuation brought by the LC film in the dynamic phantom is related to the control voltage of the film. Note that the attenuation and attenuation coefficient of an LC film of thickness *d* at a control voltage of *V* are *A_LC_* and *μ_LC_*, respectively, and then the equivalent attenuation characteristics of the film can be expressed as Eq. 3.

$$A_{\mathrm{LC}}\left(V\right)=\mu_{\mathrm{LC}}\left(V\right)\cdot d$$ (3)

After adding the film to the top surface of the basic phantom, the total attenuation seen at the DO is given by:

$$A=A_{0}+A_{\mathrm{LC}}\left(V\right)=\mu_{\mathrm{eff}0}\cdot r+\mu_{\mathrm{LC}}\left(V\right)\cdot d=\mu_{\mathrm{eq}}\cdot r$$ (4)

where *μ_eq_* is the equivalent effective attenuation coefficient, which is expressed as

$$\mu_{\mathrm{eq}}=\mu_{\mathrm{eff}0}+\mu_{\mathrm{LC}}\left(V\right)\cdot d/r$$ (5)

Equation 5 is an equivalent model of the basic optical properties of the phantom, representing the relationship between the control voltage and the equivalent light extinction coefficient. When simulating the static attenuation of a tissue with this phantom, the static optical properties can be simulated according to Eq. 5. The corresponding static control voltage could be calculated to simulate the desired static light attenuation property. In general, the structure of biological tissues does not change a lot throughout the testing process, but the concentration of substances in the tissues changes in real time with physiological activities, and the result is reflected in the optical properties of the tissue as follows: the scattering coefficient of the tissue remains basically constant as scattering is related to the tissue’s structure, but the absorption coefficient will change with the concentration of substances as the substances are the tissue’s chromosomes. Concentrations of substances are closely related to brain activities; thus, this study explores the changes in concentrations and absorption coefficients. Then, if it is assumed that all changes in the equivalent extinction coefficient of the phantom are caused by changes in the absorption coefficient, we can obtain Eq. 6,

$$\begin{array}{l} \mu_{\mathrm{eq}}=\sqrt{3\left(\mu_{a0}+\Delta \mu_{a,\mathrm{eq}}\right)\left(\mu_{\mathrm{so}}^{\prime }+\mu_{a0}+\Delta \mu_{a,\mathrm{eq}}\right)}\\ \approx \mu_{\mathrm{eff}0}+K\cdot \Delta \mu_{a,\mathrm{eq}}\left(V\right) \end{array}$$ (6)

where

$$K={\left.\frac{\partial \mu_{\mathrm{eq}}}{\partial \mu_{a}}\right|}_{\mu_{a0}}=\frac{1}{2}\sqrt{\frac{3{\mu^{\prime }}_{s0}}{\mu_{a0}}}$$ (7)

By comparing Eq. 5 and Eq. 6, we can obtain

$$K\cdot \Delta \mu_{a,\mathrm{eq}}\left(V\right)=\mu_{\mathrm{LC}}\left(V\right)\cdot d/r$$ (8)

It is further obtained that the equivalent absorption coefficients produced by the LC film to the phantom at different voltages are

$$\mu_{a,\mathrm{eq}}\left(V\right)=\mu_{a0}+\Delta \mu_{a,\mathrm{eq}}\left(V\right)=\mu_{a0}+\mu_{\mathrm{LC}}\left(V\right)\cdot \frac{d}{K\cdot r}$$ (9)

**Fig. 2.**
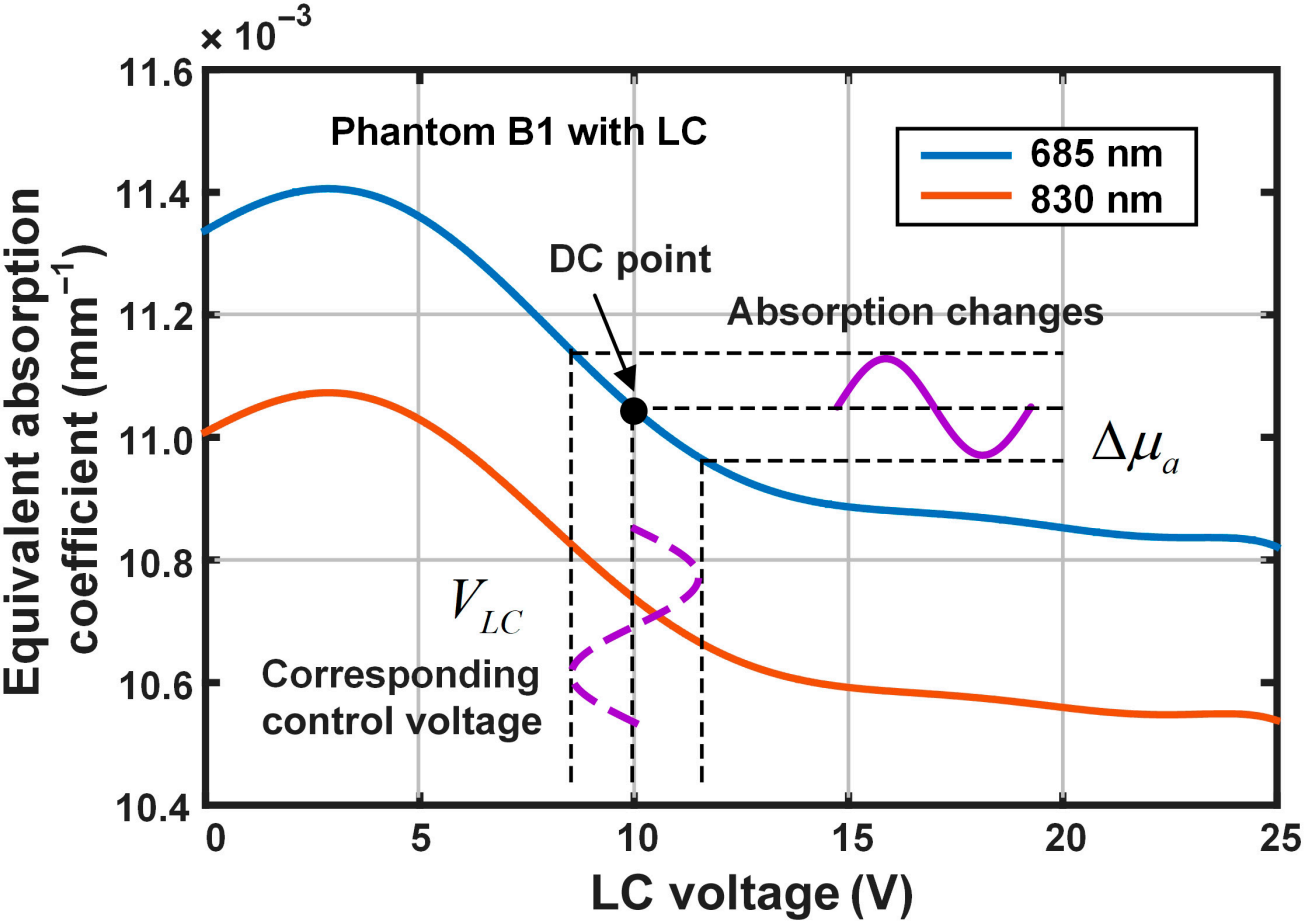
Relationship between equivalent absorption coefficient and control voltage of the designed dynamic phantom.

The relationship between equivalent absorption coefficient versus control voltage for the dynamic phantom consisting of the basic phantom layer and LC film can be obtained according to the above equation. When the SO and DO spacing is 25 mm, the equivalent absorption coefficient versus control voltage is as shown in Fig. 2. The control voltage in the range of 0 V to 25 V can make the equivalent absorption coefficient of the phantom change by 5.83×10^−4^ mm ^−1^ and 5.33×10^−4^ mm ^−1^ at 685 nm and 830 nm, respectively. By changing the control voltage around a certain static voltage point, the equivalent absorption coefficient of the phantom can also change in the same proportion. The brain NIRS signal simulation with the dynamic phantom is based on this property.

### NIRS signal model for the dynamic phantom

NIRS signal is the combination of several substances’ concentration changes. As mentioned above, the equivalent absorption coefficient of the dynamic phantom is regulated by the control voltage of the film and the equivalent absorption coefficient versus the control voltage curve is monotonically decreasing in the range of 3 V to 15 V. Therefore, if the absorption coefficient fluctuates in a small range, a linear relationship can be used to characterize the relationship between the change in control voltage and the change in absorption coefficient. Around the static control voltage *V*_0_, if the control voltage changes Δ*V*, the corresponding equivalent absorption coefficient change can be expressed as Eq. 10.

$$\Delta \mu_{a}\left(\Delta V\right)=\alpha \cdot \Delta V$$ (10)

Assuming that only one substance’s concentration is changed in the tissue, the change in absorption coefficient is therefore related to the change in concentration (Δ*C*), that is, *ε* · Δ*C* [26], where *ε* is the molar extinction coefficient of the substance. Therefore, the same equivalent Δ*μ_a_* can be produced by adjusting the control voltage of the dynamic phantom when simulating a change in the concentration of this substance. Thus, the relationship between the voltage of the dynamic phantom and substance concentration can be obtained by combining Eq. 10.

$$\varepsilon \cdot \Delta C\left(t\right)=\alpha \cdot \Delta V\left(t\right)$$ (11)

Equation 11 gives the dynamic model of the phantom in simulating the change of concentration of a single substance. If a dynamic phantom is needed to simulate the concentration change of a single substance, the corresponding control voltage for the LC film can be obtained from this equation. The equation can also be used to derive the equivalent substance concentration change for a given control voltage. If the concentrations of multiple substances are changing in the tissue under test, the timing of the film control voltage needs to be considered based on the multiplexing method used in multi-wavelength measurement.

## Design and Implementation of Optical Phantom

The basic layer of the optical phantom consists of 2 parts: the base material and the dopants. The base material is the main material of the basic layer, and the adjustment of the absorption and scattering coefficients of the basic layer is achieved by adding different absorbing and scattering extinction dopants to the base material. The main process of the basic layer can be divided into 3 steps: preparation of dopants’ solutions, phantom casting and curing, and phantom surface processing. On top of the previously fabricated basic layer, a voltage-controlled LC film is attached to adjust the optical characteristics of the phantom. This is done so as to create a complete optical phantom.

### Basic layer process

In this work, epoxy resin with high transparency and light stability was used as the base material for the optical phantom basic layer. The epoxy resin is from Alec Company, model epoxy resin glue-6600A/B. The curing of the resin can be achieved by mixing the resin (Glue A) and curing agent (Glue B) in the ratio of 3:1.

For scattering extinction dopant, titanium dioxide has the advantage of non-toxicity, stability, and protection of resin from ultraviolet damage. The scattering extinction dopant is from J&K Scientific Company, model Titanium (IV) oxide. For absorbing extinction dopants, nanocarbon powder has the advantages of small particle size and wide absorption spectrum, which can be more uniformly distributed in the resin. The absorbing extinction dopant is from Kelley Metallurgical Company, model Nano C.

The following are details of the 3 steps in the process.

**Fig. 3.**
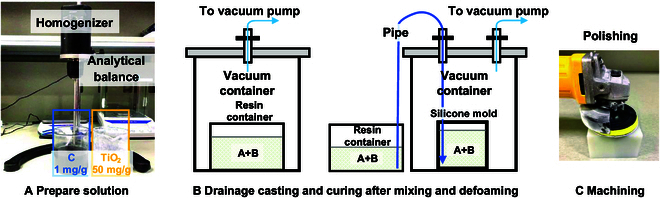
Preparations of dopants’ solutions. (A) Prepare solution. (B) Drainage casting and curing after mixing and defoaming. (C) Machining.

1. Preparations of dopants’ solutions. Quite a few dopants are sufficient to manufacture a phantom with similar *μ_a_* and $\mu_{s}^{\prime }$ to brain tissue. Reducing the error of dopant concentration is very important. In order to facilitate the weighing of the desired dopant, the dopant diluent solution was configured to minimize the error introduced by the weighing of the dopant mass. The TiO _2_ powder and C powder diluent were configured by using Glue B as the dilution liquid. The concentrations of the 2 were 50 mg/g and 1 mg/g, respectively. An analytical balance was used to reduce the weighting error. To ensure the homogeneity of the dilutions, an electric stirring homogenizer was used to fully stir the dilutions after pouring the powder to form a homogeneous suspension solution, as shown in Fig. 3A.

2. Phantom casting and curing. In order to make the basic phantom embryo, the resin glue is cured inside a square silicone mold. This mold serves as a container for curing the resin glue. The epoxy resin glue used in this work is to be cured by mixing glue A and glue B with thorough stirring in the ratio of 3:1 by weight. Air bubbles will be present in the well-mixed liquid mixture, and the presence of air bubbles will cause unwanted inhomogeneity in the light medium of the phantom and will bring a great deal of influence on light propagation. After extensive experiments, this work adopts a vacuum defoaming operation and a vacuum drainage casting mold operation for obtaining a bubble-free phantom. In the operation, the mixed liquid is first placed in a high-quality beaker with hard texture, tight structure, and smooth surface for vacuum defoaming, and then the liquid is introduced into the mold located in the vacuum barrel through the guide tube by means of vacuum negative pressure diversion, thereby completing the whole casting process. The operation diagram of mixing, vacuum defoaming, and vacuum drainage casting mold in this step is shown in Fig. 3B.

3. Phantom surface machining. In order to reduce the location difference of the optical optode on the phantom surface and improve light coupling efficiency and the consistency of the test in the meantime, it is necessary to obtain a smooth surface, regular shape, and uniform size of the phantom basic layer. The cured phantom is first cut into a rectangular shape of 90 mm in length, 90 mm in width, and 60 mm in height using a milling machine. Then, the 6 sides of the rectangular shape are polished with a polishing machine. The polishing process needs to be carried out step by step. First, the surface is roughly smoothed with 600-grit sandpaper, then the surface is polished step by step with 1,000-grit and 2,000-grit sandpaper, respectively, and lastly, the polished surface of the phantom is finely polished again with wool felt. Finally, the phantom with a smooth surface is obtained. The machining process is shown in Fig. 3C.

### Method for implementing the LC thin film layer

**Fig. 4.**
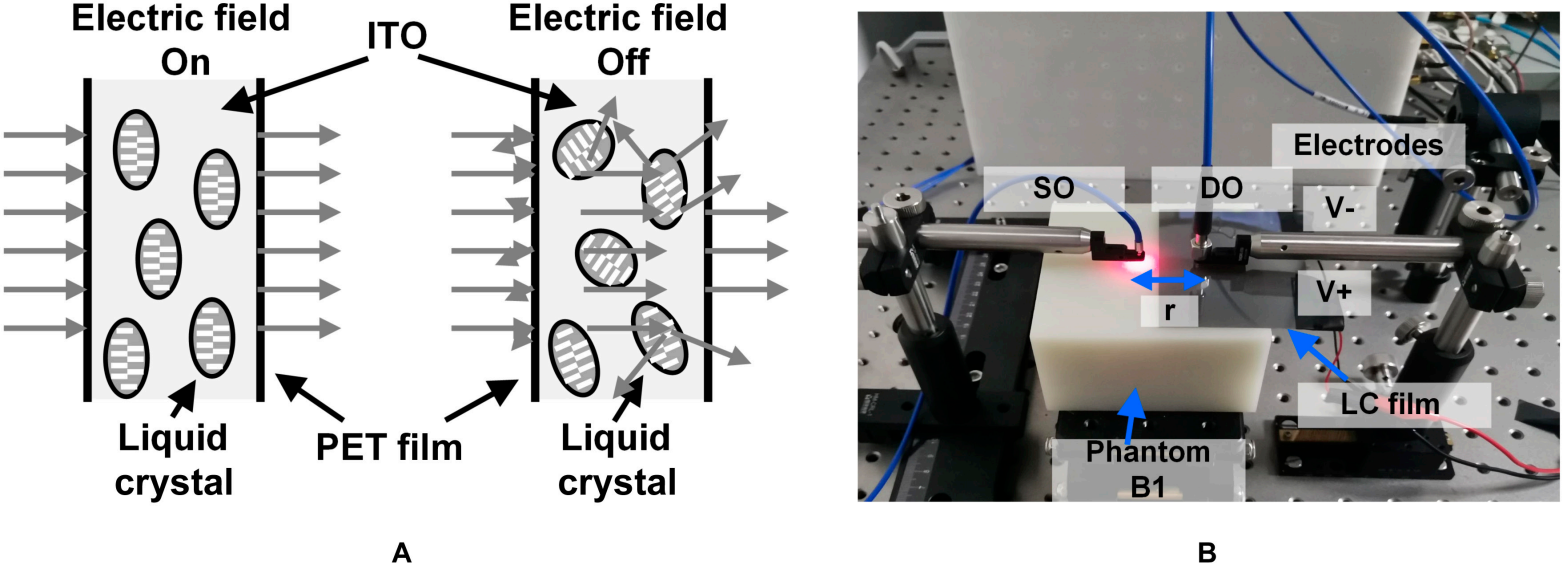
Diagram of the liquid crystal (LC) film structure and the designed dynamic phantom. (A) Structure and principle of the LC thin film. (B) Designed dynamic phantom with the LC thin film.

The core principle of the voltage-controlled LC dimming film is the electro-optical effect of LC, in which the consistency of the orientation of LC molecules can be regulated by the electric field, thus changing the optical properties of the film. The structure of the film and the constructed dynamic phantom are shown in Fig. 4. For LC films, LC molecules with controllable scattering characteristics are uniformly distributed in the flexible transparent conductive indium tin oxide (ITO) carrier, which forms the core layer. A polyethylene terephthalate (PET) plastic cover film on the outside of the core layer protects the LC to increase the lifetime of the voltage-controllable LC dimming film.

The equivalent characteristics of the LC film to the phantom need to consider the relative position within the light SO, the DO, the basic phantom, and the film, where the choice of the film position will directly affect the performance of the dynamic phantom.

Typically, there are 3 positions to locate the LC film. The first is inside the basic phantom, the other 2 are on the surface of basic phantom, in front of either SO or DO. The LC film scatters light photons rather than absorbing them; thus, when the LC film is inserted inside a highly scattering medium, the scattering effect by the film is relatively weak compared to the effects of a basic phantom layer, and therefore, the dynamic range of adjustable optical characteristics is quite limited. According to the measured results, the dynamic range of the emergent light intensity detected by the DO is less than 0.5% when the thin film is inserted in the phantom at an source–detector (SD) spacing of 25 mm, which can only simulate the NIRS signal within a small amplitude, making it difficult to simulate the NIRS signal comprehensively and accurately. The situation will change when the film is located on the surface of the basic phantom layer in front of the DO, and the light intensity received by the DO can be regulated directly. The dynamic ranges increase to more than 10%. Another advantage of placing the film on the DO side and not the SO side is its independence when used with a multi-channel NIRS device. A large-scale optode array is used in multi-channel NIRS devices, which contain many SOs and DOs. The large-scale optode array generally employs time-division multiplexing to control the duty timing of each light source. If each DO is equipped with an independently controllable film and combined with the SO time-division multiplexing timing, the dynamic phantom can independently control the light intensity of each measurement channel, which is an important function that cannot be achieved by placing the film in front of the light SO as the light emitted by one SO will be detected by many DOs.

**Fig. 5.**
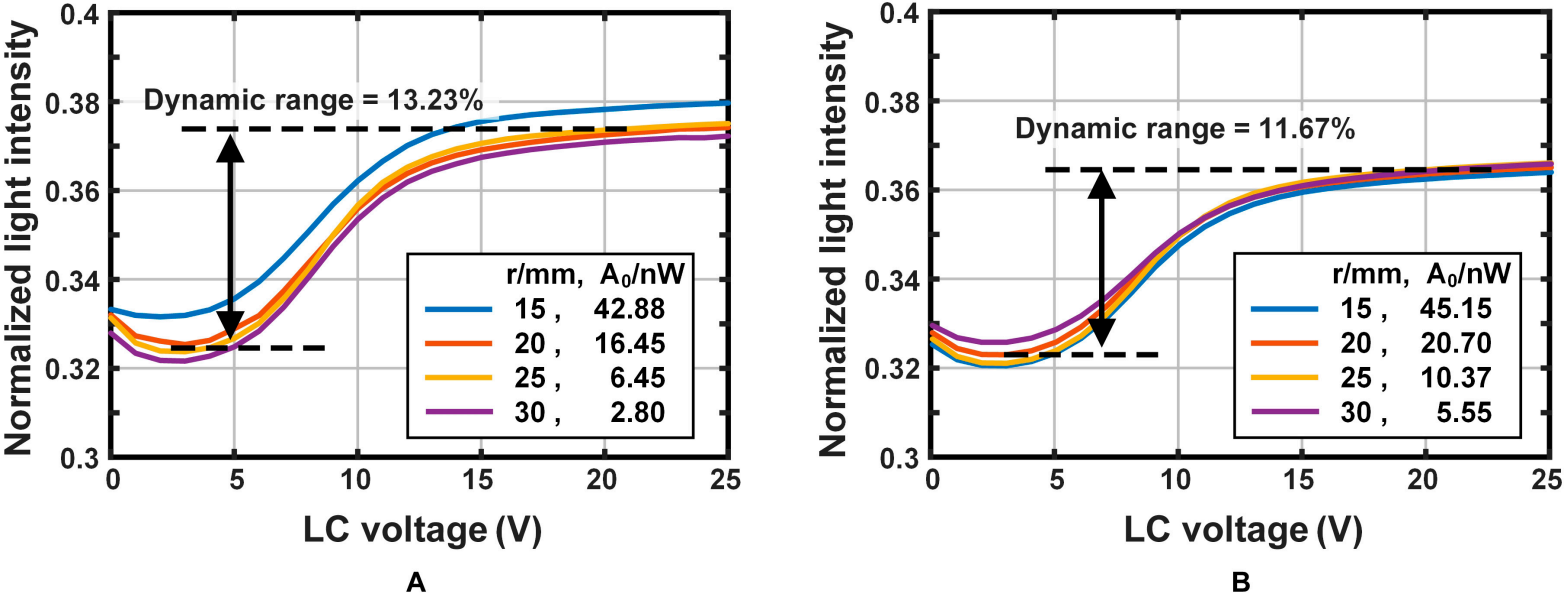
Detected light power of detect optode versus control voltage of the liquid crystal film. (A) Wavelength 685 nm. (B) Wavelength 830 nm.

Therefore, in this work, the optical phantom is constructed with a thin film attached to the top surface of the phantom at the surface of the detection optical optode. The film was fixed on the top surface of the phantom, and a single-core optical fiber with a core diameter of 1 mm and a numerical aperture of 0.37 was used as the DO to measure the effect of the film on the light intensity of the DO at an SD spacing *r* from 15 mm to 30 mm with a test interval of 5 mm and test wavelengths of 685 nm and 830 nm, respectively. The test results are shown in Fig. 5, from which it can be seen that the LC film achieves the regulation of the light intensity received by the DO. When no electric field is applied, the film makes the light intensity received by the DO decrease to about $\frac{1}{3}$, and with the increase of voltage, the received light intensity gradually increases and reaches a steady state. The film has different adjustment strengths for different wavelengths of light. For 685-nm and 830-nm light, the maximum dynamic adjustment range of light intensity achievable by the films is 13.23% and 11.67%, respectively, which can basically cover the range of light intensity amplitude variation corresponding to the NIRS signal. The light intensity regulation ranges at different SD separations are almost the same. The reason is that the morphology of the light emitted from the top surface at different positions is the same. Therefore, it can be regarded as an isotropic uniform light source in the field of view of the fiber. In addition, the relative positions of the fiber, the film, and the top surface are the same, which ensures the consistency of the dynamic adjustment characteristics when the optodes are placed at different positions on the phantom top surface. Therefore, placing the thin film in front of the DO can realize an independently controllable optical phantom with a large dynamic range.

## Results

The NIRS signal is composed of changes of the oxy-/deoxy-hemoglobin concentrations. In order to evaluate the simulation capability of the phantom, the simulation error of the phantom to mimic each kind of hemoglobin concentration change at different wavelengths was first tested, and then the effect of the phantom when simulating a typical NIRS signal was measured.

The spacing between the light SO and the DO was 25 mm, and the static control voltage of the dynamic phantom was set to 10 V at both 685 nm and 830 nm, where the equivalent absorption coefficient of the phantom was roughly at the middle point of the regulating range. According to Fig. 2, the equivalent absorption coefficients were 11.56 × 10^−3^ mm^−1^ and 11.42 × 10^−3^ mm^−1^ when the static control voltage was 10 V, and the absorption coefficient–voltage sensitivity of the dynamic phantom was calculated to be −0.60 × 10^−4^ mm^−1^/V and −0.55 × 10^−4^ mm^−1^/V. The molar extinction coefficients at the 2 wavelengths are 6.30 × 10^−5^ and 2.24 × 10^−4^ mm^−1^/μmol/l for oxy-hemoglobin and 5.12 × 10^−4^ and 1.60 × 10^−4^ mm ^−1^/μmol/l for deoxy-hemoglobin, respectively. According to Eq. 11, the corresponding control voltages can be obtained when these 2 hemoglobin concentrations vary in the range [−0.5 μmol/l, 0.5 μmol/l].

**Fig. 6.**
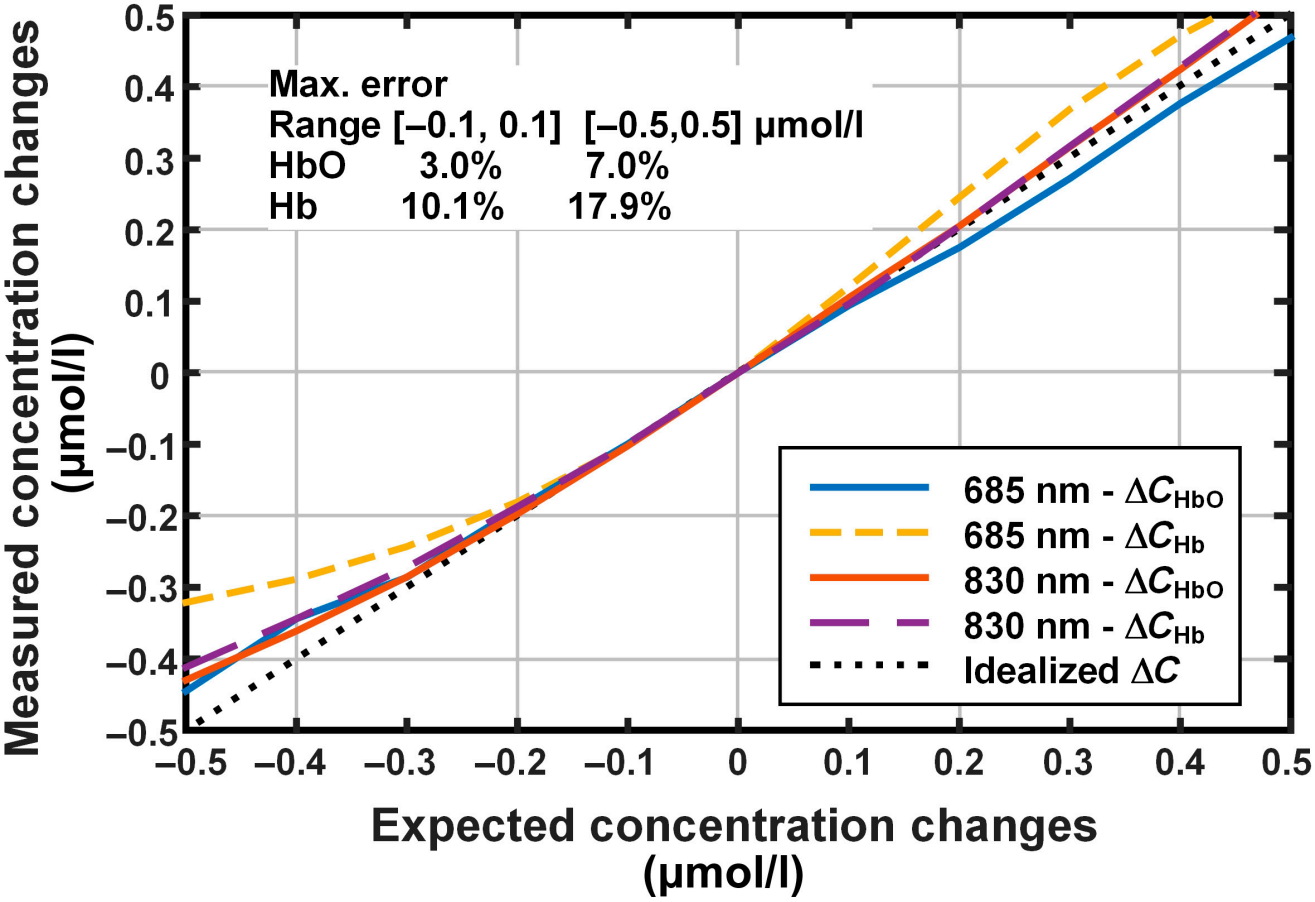
Results of oxy-/deoxy-hemoglobin concentration change simulation with the dynamic phantom.

In order to mimic the equivalent substance concentration changes at each wavelength, we applied the appropriate voltage to the LC film and resolved the detected light intensity using MBLL. The results are as shown in Fig. 6. As can be seen from the figure, when the simulated concentration range is ±0.1 μmol/l, the simulated nonlinear errors are only 3.0% and 10.1% for oxy-hemoglobin and deoxy-hemoglobin, respectively. When the simulated range is expanded to ±0.5 μmol/l, the simulated nonlinear errors for both substances then increase to 7.0% and 17.9% (685 nm), and 7.0% and 8.8% (830 nm), which extends the dynamic simulation range by more than an order of magnitude compared to the similar solid-state dynamic phantom [11]. What needs to be mentioned is that a linear approximate model is used in the method, and the simulation error is mainly dependent on the linearity of the phantom extinction ability versus control voltage, which is related to the extinction of oxy-/deoxy-hemoglobin. The largest error appears on the deoxy-hemoglobin concentrate at 685 nm, where the linearity error of the phantom is amplified by the large extinction coefficient of deoxy-hemoglobin at 685 nm, which is much larger than the others.

**Fig. 7.**
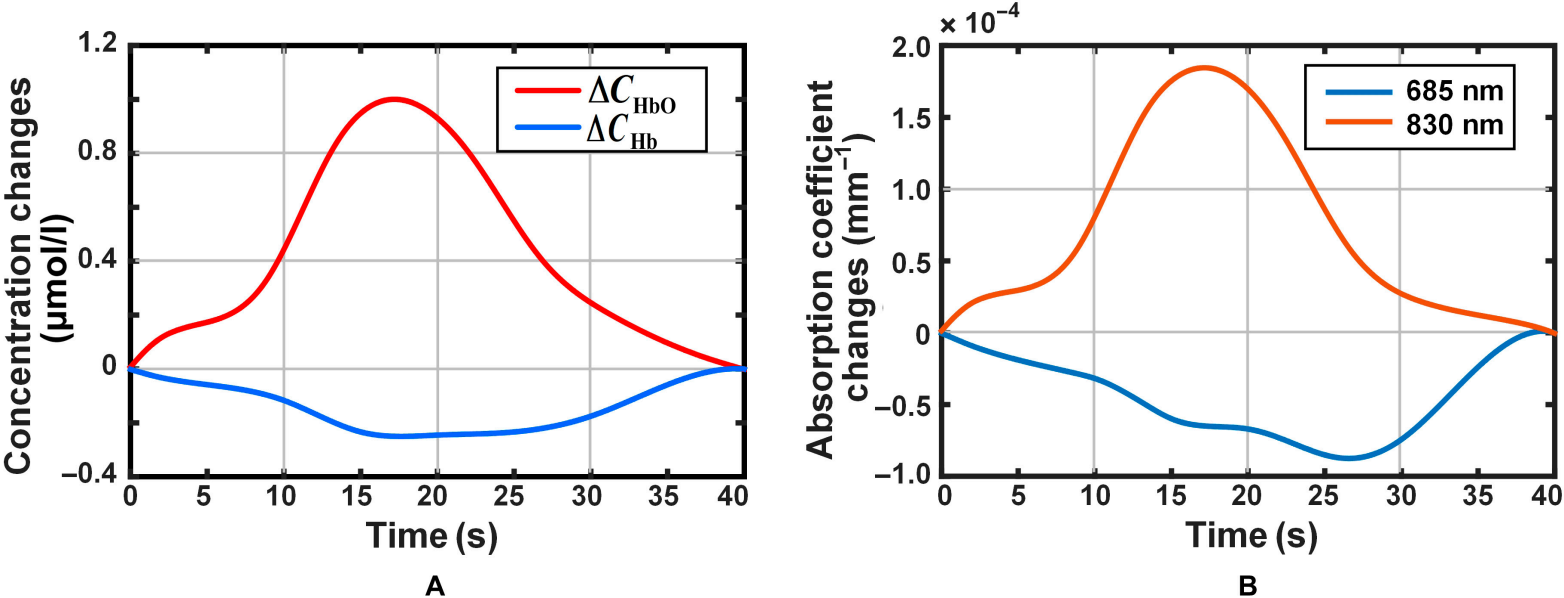
Results of NIRS signal simulation with the dynamic phantom. (a) Idealized NIRS signal. (b) Expected corresponding absorption coefficient changes.

In order to verify the ability to simulate the NIRS signal, an ideal NIRS signal (Fig. 7A) was simulated using the designed phantom and the established linear voltage model. Using the relationship between substance concentration and absorption coefficient, the corresponding absorption variations at 685-nm and 830-nm wavelengths are shown in Fig. 7B. It is observed from this figure that the maximum change in absorption coefficient is −0.88 ×10^−4^ mm ^−1^ and 1.85 ×10^−4^ mm ^−1^, respectively, which are within the regulating range of the equivalent absorption coefficient of the phantom.

**Fig. 8.**
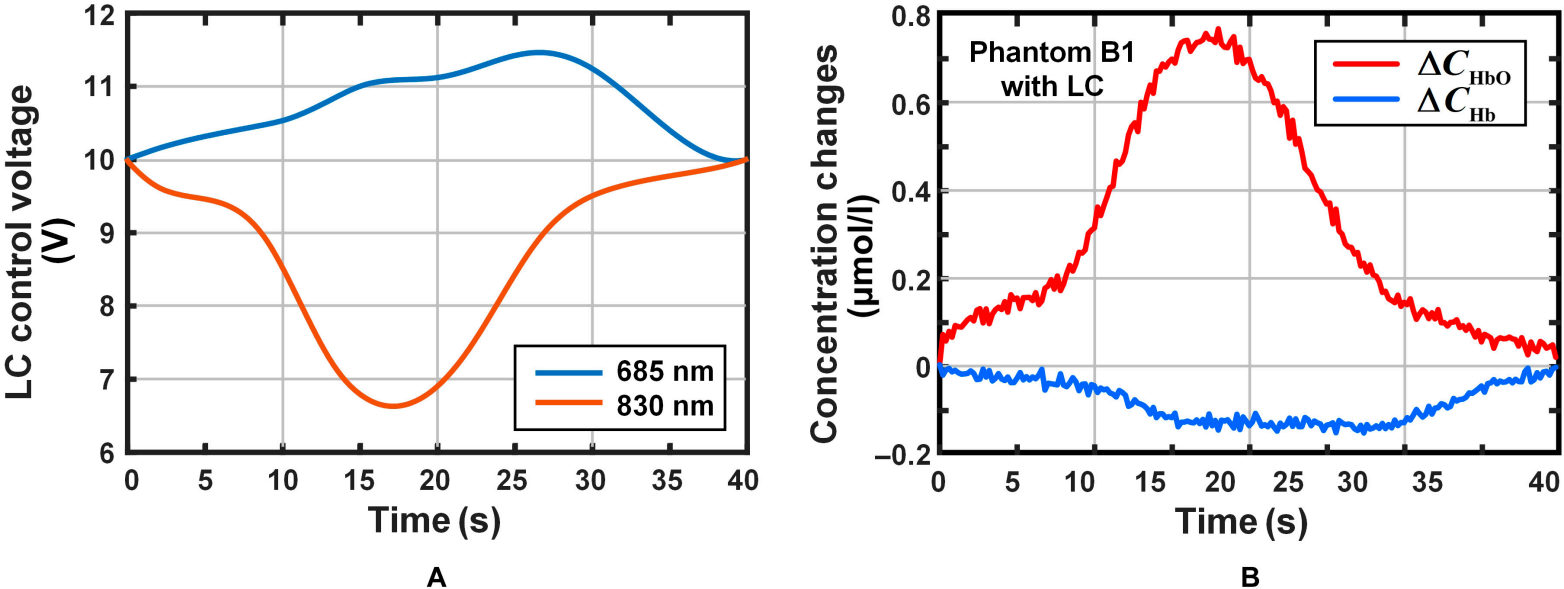
Results of NIRS signal simulation with the dynamic phantom. (A) Corresponding control voltage of the liquid crystal thin film. (B) Measured concentration changes of oxy- and deoxy-hemoglobin.

The control voltage variation curves of LC films at different wavelengths are shown in Fig. 8A. The corresponding voltage variation ranges from 10 V to 11.46 V when the wavelength is 685 nm and from 10 V to 6.63 V when the wavelength is 830 nm. The corresponding control voltages are sampled at 10 Hz according to the time sequence of time division multiplexing of the dual-wavelength light source. Then, the final film control voltage time series is generated for regulating the change in the equivalent absorption coefficient of the dynamic phantom to simulate the expected NIRS signal. The dynamic phantom was tested using a signal channel NIRS device. During the test, the SD spacing is 25 mm apart, and the LC film is regulated by the control voltage. MBLL is used to resolve the light intensity received by the DO to obtain the changes in the oxy-hemoglobin and deoxy-hemoglobin concentrations, as Fig. 8B shows. The calculated variation curves of the 2 hemoglobin concentrations (measured NIRS signals) were compared with those ideal NIRS signals. According to the results of the measurements, the mimicked NIRS signals were basically consistent with the waveform shape and the variation trend of the ideal NIRS signals, and less pronounced than the ideal waveform in waveform amplitude, which resulted in the maximum error for the 2 hemoglobins being 0.13 μmol/l and 0.10 μmol/l, respectively.

## Conclusion

In this work, a novel voltage-controlled optical dynamic phantom for brain NIRS signal simulation is constructed, and an optical equivalent model of the phantom’s adjustable optical properties versus control voltage is built. Through the reasonable selection of the phantom fabrication materials and the optimization of the processing and fabrication process, a phantom basic layer with a regular shape and smooth surface is finally obtained. On the top surface of this, a voltage-controllable LC film is fixed in the front end of the DO to regulate the light directly. Depending on this solid–solid dynamic phantom, equivalent light extinction properties and NIRS signals could be regulated by voltage conveniently. The constructed dynamic phantom has a higher fast regulation rate and a broader dynamic range than conventional phantoms, which can be used to simulate most brain NIRS signals. Based on the proposed solid dynamic phantom, the performance of the NIRS device could be verified sufficiently; in particular, the high stability of the phantom is an important factor in quality assurance measurement to receive Food and Drug Administration approval, and with the ability of mimic NIRS signals, the phantom can also be used as a head phantom in NIRS operating training. All of these applications will benefit the promotion of NIRS in BCI application.

## Data Availability

The data are freely available upon request.
